# Histamine‐receptor blockade does not influence the heavy‐severe domain boundary and time to task failure in the severe domain during cycling exercise in adults

**DOI:** 10.14814/phy2.70587

**Published:** 2025-09-29

**Authors:** Kieran S. S. Abbotts, Jake H. Hudgins, Isabella S. Viveros, Christopher T. Minson, Brad W. Wilkins, John R. Halliwill

**Affiliations:** ^1^ Department of Human Physiology University of Oregon Eugene Oregon USA

**Keywords:** allergies, bicycling, exercise performance, exercise thresholds, fatigue, histamine, physiology

## Abstract

The influence of histamine in skeletal muscle during exercise is poorly characterized. This investigation tested the hypothesis that histamine‐receptor blockade lowers the power associated with the heavy‐severe domain boundary and reduces time to task failure in the severe domain. Following a graded exercise test and a familiarization trial, 17 participants (8 M/9 F, 29 ± 8 years, VO_2peak_ 60.0 ± 7.5 mL/kg/min, mean ± SD) completed cycle ergometer exercise on two separate occasions, after either histamine‐receptor blockade or placebo, in a double‐blind randomized crossover protocol. Exercise intensities were designed to span the moderate, heavy, and severe domains. Skeletal muscle tissue oxygen saturation (%SmO_2_, via near‐infrared spectroscopy) and expired gases were measured continuously throughout exercise. There were no differences between blockade and placebo in power associated with the heavy‐severe domain boundary (216 [195, 236] vs. 213 [191, 234] W, mean [95% CI]; *p* = 0.41) or time to task failure (474 [377, 572] vs. 473 [380, 566] s; *p* = 0.95). %SmO_2_ slope decreased, and oxygen uptake increased with intensity (*p* < 0.01), but were not affected by blockade (all *p* > 0.05). These findings suggest that histamine is not crucial to supporting power at the heavy‐severe domain boundary or short‐duration exercise in the severe domain.

## 1 INTRODUCTION

Traditionally, histamine has been viewed through the context of allergies and inflammation. However, during exercise, histamine is released in exercising skeletal muscle, where it can act in an autocrine and paracrine fashion by binding to H_1_ and H_2_ receptors located on immune and vascular cells within the muscle environment (Halliwill et al., 2013; Luttrell & Halliwill, 2017; Romero, McCord, et al., 2017; Romero, Minson, & Halliwill, 2017; Van Der Stede et al., 2025). Histamine is the primary mediator of sustained postexercise vasodilation, evidenced by H_1_ and H_2_ receptor blockade abolishing postexercise increases in vascular conductance and reducing postexercise hypotension (Lockwood et al., 2005; McCord et al., 2006; McCord & Halliwill, 2006). Blocking its actions reduces sustained postexercise vasodilation (Barrett‐O'Keefe et al., 2013; McCord et al., 2006; McCord & Halliwill, 2006), alters the transcriptional (Romero et al., 2016) and inflammatory response to exercise (Van Der Stede et al., 2025), and attenuates functional adaptations to exercise training (Sieck et al., 2025; Van der Stede et al., 2021). Clearly, histamine is important in the postexercise period, facilitating recovery from and adaptation to exercise, but its role during exercise is poorly characterized.

Initial investigations into histamine's role in exercise performance found that in trained cyclists, histamine‐receptor blockade slows 10‐km time trial performance (Ely et al., 2019). For perspective, this degree of impaired performance is enough to negate the ergogenic benefit of caffeine (Guest et al., 2018) and can be the difference between first place and off the podium (Currell & Jeukendrup, 2008). Given the importance of histamine in the postexercise blood flow response, it was suggested that histamine‐receptor blockade worsened performance by reducing blood flow to exercising skeletal muscle (Barrett‐O'Keefe et al., 2013). However, a follow‐up study found that histamine‐receptor blockade paradoxically increases femoral blood flow during unilateral knee extension exercise while also resulting in greater decreases in intramuscular pH (Ely et al., 2020). These divergent findings illustrate a “histamine paradox,” as increases in femoral blood flow should theoretically attenuate decreases in intramuscular pH and improve performance. A potential explanation may lie in histamine's role as an intrinsic regulator of the microvasculature (Majno et al., 1969; Schayer, 1962). It is possible that histamine‐receptor blockade alters the distribution of blood flow within skeletal muscle, independent of “bulk” blood flow measures in conduit arteries, impacting nutrient delivery and metabolite washout. This may also affect the balance of oxygen supply and demand in exercising skeletal muscle, which is important in exercise performance.

The sustainability of a given exercise intensity can be determined by whether or not it falls above or below the heavy‐severe domain boundary (Jones et al., 2019). Exercise below this boundary (heavy domain) results in steady state oxygen uptake and muscle metabolite responses, whereas above this boundary (severe domain), individuals cannot achieve a steady state, resulting in the attainment of maximal oxygen uptake and/or ultimately task failure (Jones et al., 2019). The physiology describing this boundary is the maximal metabolic steady state (Jones et al., 2019), which is the highest intensity where oxygen supply and demand are balanced (Kirby et al., 2021).

Traditionally, this has been described by evaluating measurements of power, lactate, or expired gases to determine whole body thresholds, such as critical power, maximal lactate steady state, and respiratory compensation point. These whole body thresholds are better able to identify the maximal metabolic steady state than local measures such as deoxygenated hemoglobin, myoglobin breakpoint, and the breakpoint of the integrated electromyography signal (Caen et al., 2022). However, these methods all have limits as they require expensive laboratory equipment, blood sampling, or exhaustive exercise trials. Evaluating dynamic changes in muscle oxygenation (%SmO_2_) using commercially available portable near‐infrared spectroscopy (NIRS) devices has emerged as a viable alternative to evaluate the maximal metabolic steady state. By evaluating the changes in %SmO_2_ over time (%SmO_2_ slope) during varying workloads, the highest workload at which oxygen supply matches demand, that is, a zero %SmO_2_ slope, can be determined. This methodology accurately identifies the maximal metabolic steady state (Kirby et al., 2021; Matthews et al., 2023; Wilkins et al., 2025) and has excellent reliability (ICC > 0.9) and low measurement error (<2%) for predicting power at the heavy‐severe domain boundary for both men and women (Hudgins et al., n.d.).

To our knowledge, only one study using single‐leg knee extension tested the impact of histamine‐receptor blockade on %SmO_2_ using NIRS; however, the investigators did not assess the %SmO_2_ slope as it pertains to identifying the maximal metabolic steady state. It is unknown if the maximal metabolic steady state is altered by histamine‐receptor blockade (Ely et al., 2020). The primary aim of this study was to investigate the impact of histamine‐receptor blockade on work rate associated with balanced muscle oxygen supply and demand (i.e., zero slope). We hypothesized that combined H_1_ and H_2_ receptor blockade would lower the power associated with the heavy‐severe domain boundary when compared to placebo. In addition, we aimed to investigate the impact of histamine‐receptor blockade on cycling performance as measured by time to task failure in the severe domain. We hypothesized that blockade would decrease the time to task failure in the severe domain when compared to placebo.

## 2 MATERIALS AND METHODS

### 2.1 Participants

Seventeen healthy, nonsmoking individuals (8 males, 9 females, self‐reported) participated in this study. Individuals were between the ages of 18 and 60 who were experienced in cycling and classified as “trained” or “well‐trained” (Decroix et al., 2016; Pauw et al., 2013). Participant characteristics are outlined in Table 1.

**TABLE 1:** Participant characteristics.

Variable	All	Male	Female	*p* Value
Age (years)	29 ± 8	30 ± 10	29 ± 5	0.706
Height (cm)	172 ± 9	178 ± 9	166 ± 5	0.004
Weight (kg)	66.0 ± 8.2	72.8 ± 6.6	60.0 ± 3.2	<0.001
BMI (kg/m^2^)	22.4 ± 1.6	23.0 ± 1.4	21.8 ± 1.6	0.112
VO_2peak_ (L/min)	3.98 ± 0.80	4.70 ± 0.45	3.33 ± 0.37	<0.001
VO_2peak_ (mL/kg/min)	60.0 ± 7.5	64.8 ± 5.7	55.7 ± 6.4	0.008
Peak power (W)	333 ± 51	378 ± 28	292 ± 26	<0.001
Relative peak power (W/kg)	5.03 ± 0.41	5.21 ± 0.33	4.88 ± 0.44	0.106
*n*	17	8	9	

*Note*: Peak power is the highest 30‐s power during the graded exercise test. The *p* value represents differences between males and females. Data are mean ± SD.

### 2.2 Experimental design

**FIGURE 1:**
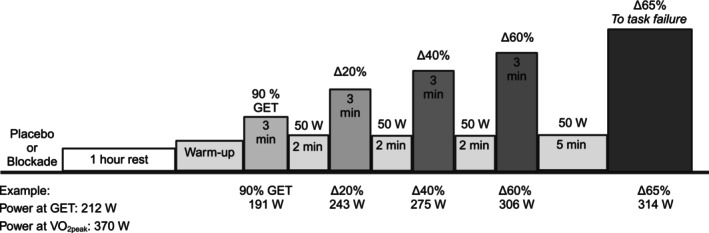
Schematic of the testing visits. Participants ingested placebo or antihistamines 1 h before exercise. Participants completed a 10‐min self‐paced warm‐up and then were instrumented with a mouthpiece for the collection of expired gases. Participants then completed a 10‐min standardized warm‐up immediately followed by the first stage (90% GET, gas exchange threshold). Example power outputs are based on data from a single participant. Created in BioRender.

The study consisted of four laboratory visits, including screening, familiarization, and two experimental testing sessions. The screening visit consisted of obtaining informed consent, measuring demographic and anthropometric information, and conducting a graded exercise test to determine peak oxygen uptake (VO_2peak_). Provided they met the inclusion criteria, participants then reported to the laboratory on three separate occasions: one familiarization visit and two double‐blind, placebo‐controlled testing visits. The testing visits (i.e., blockade or placebo) occurred in a randomized order and were separated by a minimum of 1 week. A schematic of the testing visits is presented in Figure 1. Primary outcomes were the power associated with the heavy‐severe domain boundary, as determined by the zero‐slope method, and the time to task failure in the severe domain. Secondary outcomes were %SmO_2_, heart rate, rating of perceived exertion, and expired gas measures.

### 2.3 Measurements

The graded exercise test and all subsequent exercise sessions were conducted using an electronically braked cycle ergometer (Lode Excalibur Sport, Groningen, The Netherlands) and took place in a temperature‐controlled laboratory (~20°C). The ergometer seat and handlebars were adjusted to the cyclist's specifications and replicated for all exercise sessions. Heart rate was measured via telemetry (Model H10, Polar Electro, Kempele, Finland) throughout all exercise bouts. Oxygen uptake (VO_2_) and carbon dioxide production (VCO_2_) were measured by indirect calorimetry using a mixing‐chamber system (Parvo Medics, TrueOne 2400 Sandy, UT). Expired gas values are averages from the final minute of the stage unless otherwise noted.

During the familiarization and testing visits, an index of muscle tissue saturation (%SmO_2_) was recorded continuously via NIRS (Moxy Monitors, Fortiori Design LLC, Hutchinson, MN) based on the ratio of oxyhemoglobin and myoglobin concentration to the total hemoglobin and myoglobin concentration in the tissue and expressed as a percentage. When used as specified, %SmO_2_ is an index of tissue oxygenation at the microvascular level and is weighted to largely reflect the skeletal muscle under the sensor but with some contribution from other tissues (Barstow, 2019; Craig et al., 2017; Feldmann et al., 2019; Ryan et al., 2012). NIRS has been used previously to delineate intensity domains, identify the highest sustainable steady state during exercise, and assess recovery from an exercise bout, and its use in this capacity compares favorably to more time‐intensive approaches (Batterson et al., 2020, 2023; Iannetta et al., 2017; Kirby et al., 2021; Matthews et al., 2023). To omit any light interference, sensors were enclosed in a black light‐blocking case provided by the manufacturer and affixed with adhesive tape. For consistent sensor placement across all trials, sensors were placed on both the right and left vastus lateralis, 2/3rds the distance on the line from the anterior superior iliac spine to the lateral side of the patella, consistent with previous investigations (Batterson et al., 2020, 2023). Reported tissue oxygenation is the average over the final minute of the stage unless otherwise noted.

### 2.4 VO_2peak_ test

The ramp‐style protocol began with 3 min of cycling at 60 watts before the resistance was increased at a fixed rate until volitional exhaustion. The ramp rate (1 watt every 2 s or 1 watt every 2.4 s) was determined such that all participants would reach exhaustion within 8–12 min. The test was terminated when the subjects were unable to maintain a cadence ≥60 RPM, despite strong verbal encouragement. Following the test, participants recovered for 10 min before performing a supramaximal verification trial to ensure no further increases in peak oxygen consumption at a greater power output than that which elicited a plateau in oxygen consumption (Poole & Jones, 2017). The verification trial was performed at a constant power output of 110% of the peak power achieved during the ramp until volitional exhaustion (unable to maintain a cadence ≥60 rpm). VO_2peak_ was determined as the highest 30‐s average during the protocol, and gas exchange threshold (GET) was estimated using the V‐slope method (Beaver et al., 1986). When identifying the power output at VO_2peak_ and at GET, the lag in VO_2_ was accounted for by deducting 2/3 of the incremental ramp rate from the power output that was time aligned with VO_2peak_ and GET (Boone et al., 2008; Lansley et al., 2011; Vanhatalo et al., 2016).

### 2.5 Determination of stage exercise intensities

For each participant, the intensities for the staged exercise test were determined based on the results of the VO_2peak_ test and spanned the moderate, heavy, and severe domains. The power output corresponding to GET was determined as the transition between the moderate and heavy domains, as previously established (Black et al., 2017; Jones et al., 2011; Lansley et al., 2011). To derive exercise intensities for the heavy and severe domains, the relative difference (%∆) between the power output corresponding to GET and the power output corresponding to VO_2peak_ was determined and added to the power output corresponding to GET for each participant. This method has previously been used to accurately define moderate, heavy, and severe domain exercise (Black et al., 2017; Burnley et al., 2000; Kirby et al., 2021; Lansley et al., 2011). Since the goal was to span the heavy and severe domains, the power output corresponding to ∆50% was used to approximate the boundary between the heavy and severe domains (Black et al., 2017; Lansley et al., 2011; Matthews et al., 2023), and intensities were selected that would be appreciably above and below this “transition.”

The intensities for the staged exercise test were as follows: Stage one was at the power output corresponding to 90% of the intensity at GET (moderate domain). Stage two was at the power output corresponding to ∆20% (heavy domain), stage three was at the power output corresponding to ∆40% (heavy domain), and stage four was at the power output corresponding to ∆60% (severe domain). The time to task failure trial was completed at a power output corresponding to ∆65% (severe domain).

### 2.6 Familiarization visit

Participants performed a familiarization visit to acquaint them with the exercise and laboratory instrumentation. The visit and instrumentation were the same as the experimental testing visits apart from the ingestion of placebo or antihistamines and subsequent hour wait. The familiarization exercise test protocol was similar to the experimental visits' protocol, except the final time to task failure trial was omitted (i.e., cool‐down began after stage four). Following the visit, participants were given a 24‐h dietary recall and instructed to document their diet 24 h prior to their first testing visit.

### 2.7 Experimental testing visits

Following the familiarization visit, participants returned to the laboratory on two separate occasions, separated by at least 1 week. Each of these visits started with a confirmation of adherence to the abstention criteria and collection of the dietary recall, then body mass was obtained and participants ingested either antihistamines (540 mg fexofenadine, a selective H_1_‐receptor antagonist (Allegra 180 mg, National Drug Code 41167‐4120‐3, Sanofi, SA, Paris, France) and 40 mg famotidine, a selective H_2_‐receptor antagonist (Pepcid 20 mg, National Drug Code 16837‐855‐14, Kenvue LLC, Skillman, New Jersey, United States)) or placebo (Blue/White Capsules (manufacturer stock keeping unit number ES‐1HWN‐7EXH), White Round Tablets (manufacturer stock keeping unit number E6‐QO6L‐Y2K9), Zeebo Effect LLC, Huntington, Vermont, United States). This dosage of histamine‐receptor antagonists results in more than 90% inhibition of histamine H_1_ and H_2_ receptors lasting for 6 h after administration and is consistent with previous histamine‐receptor blockade studies (Echizen & Ishizaki, 1991; Russell et al., 1998; Van der Stede et al., 2021). Participants were blinded to the treatment they were receiving and instructed not to inspect the pills they were taking (i.e., pills were placed in their hands and ingested immediately so they were unable to observe the shape, size, and texture).

Following administration of histamine‐receptor blockade or placebo, participants rested for 1 h to facilitate absorption (Echizen & Ishizaki, 1991; Russell et al., 1998; Van der Stede et al., 2021). During this hour, participants were instrumented with a heart rate monitor and NIRS‐Moxy sensors. After approximately 1 h post‐ingestion, participants completed a self‐selected warm‐up (recorded and replicated in the subsequent exercise trial). Participants were then instrumented with a nose clip and mouthpiece to collect expired gases. Participants began a 10‐min standardized warm‐up, consisting of 30 s of cycling at each stage's intensity, separated by 30 s of cycling at 50 watts, followed by 5 min of cycling at 50 watts. Stage one of the staged exercise test began immediately following the standardized warm‐up.

The staged exercise test consisted of four 3‐min‐long stages of increasing intensity. Each stage was separated by 2 min of active recovery, during which participants cycled at 50 watts. Following stage four, participants cycled for 5 min at 50 watts before completing a final stage until task failure. Task failure was defined as either volitional exhaustion or when the participant could no longer maintain a cadence above 60 RPM despite verbal encouragement. During the final stage, participants were provided with verbal encouragement but no information regarding the time elapsed. Heart rate was continuously monitored and recorded during the last 30 seconds of each stage. Rating of perceived exertion (Borg 6–20) was recorded during the last 30 s of each stage. During the final stage, the heart rate was recorded as the highest (usually in the last 30 s), and the rating of perceived exertion was taken immediately following task failure.

### 2.8 Determination of %SmO_2_ zero‐slope

Data from the NIRS‐Moxy sensors were sampled at 2 Hz and imported into a Microsoft Excel file for analysis. For each exercise stage, %SmO_2_ was plotted against time for each stage for both the right and left legs. Previous findings (Kirby et al., 2021) observed a rapid transient onset phase resulting in an initial nadir in %SmO_2_ signal. To account for this transient response, the initial nadir was identified and omitted from the stage‐slope determination. The stage %SmO_2_ slope was determined from the linear regression of %SmO_2_ and time (in minutes) following the nadir (~60 s into the stage) until the end of the stage. If a portion of the stage contained a loss of %SmO_2_ signal, the stage slope was taken from the longest uninterrupted segment of %SmO_2_ data lasting at least 90 s (half the stage duration). If the loss of signal made it such that there was less than 90 s of uninterrupted %SmO_2_ data, that leg was omitted from the slope analysis. For the time to task failure trial, the slope was determined from the regression of %SmO_2_ and time (in minutes) following the nadir (~60 s into the stage) for the longest reliable time (at least 90 s) following the nadir.

To determine the power output that elicited a zero‐slope, the previously calculated slopes from each stage were plotted on the y‐axis against power on the x‐axis for both legs. Then, a linear regression model was performed for each leg to identify the x‐intercept—the power at which the slope equaled zero (Matthews et al., 2023; Wilkins et al., 2025). Stages were omitted from the linear regression for determining the power at zero slope if they did not contain reliable data. Zero‐slope power for the trial was determined by averaging the zero‐slope power of both legs. If one leg had a linear regression that returned an *R* ^2^ value of less than 0.85 or had less than three stages included in the regression (i.e., two or more stages omitted), it was not used to determine zero‐slope power. If the power from one leg was omitted, the zero‐slope power from the leg included was used for the visit instead of the average of both legs. The slopes of each stage and the power that elicited a zero slope were determined by two experienced, independent reviewers.

### 2.9 Rigor and reproducibility

None of the participants were using any over‐the‐counter or prescription medications at the time of the study, except for oral contraceptives. Women were studied irrespective of menstrual cycle phase and had a negative pregnancy test prior to all assessment days. The menstrual cycle was not controlled for in female participants as female athletes generally train and compete throughout all phases of the menstrual cycle, and previous research indicates that the menstrual cycle has little effect on exercise performance (D'Souza et al., 2023; McNulty et al., 2020; Taylor et al., 2024) or other exercise responses that are dependent on histamine receptor activation, such as postexercise hemodynamics (Lynn et al., 2007; Pellinger et al., 2010; Senitko et al., 2002; Shiozawa et al., 2023). Prior to all assessments, participants were asked to abstain from alcohol, strenuous exercise, over‐the‐counter medication for 24 h, and food and beverages (except water), and caffeine for 2 h. Data collection start time varied across participants due to scheduling conflicts; however, exercise testing visits for each participant were conducted at the same time of day (±1 h) to reduce the potential influence of circadian rhythms on muscle blood flow and exercise performance. Participants were asked to maintain a similar diet prior to each of their testing visits (i.e., blockade or placebo), which was confirmed via collection of a 24‐h dietary recall at each visit. All protocol activities took place in a thermoneutral lab environment. Blinding and randomization were done by an individual not involved in data collection or analysis. Investigators, including those involved in data entry and analysis, and participants were blind to the condition and order of administration.

### 2.10 Statistics

Participant characteristics are presented as mean ± SD. Given that the study was not intended to examine sex differences, only differences in characteristics between males and females were compared, and these were compared using multiple independent *t*‐tests. All other data are reported as mean with 95% confidence intervals. Alpha was set at 0.05 for all statistical inferences including familywise error rates. Shapiro–Wilks tests were used to test for normality. Inferences regarding time to task failure and estimated power at zero‐slope with blockade or placebo were drawn using a paired *t*‐test (Prism 10, GraphPad, Boston, MA, USA). Inferences surrounding variables of interest over time were drawn from two‐way mixed‐effects models (for measurements across time and between conditions) with preplanned comparisons. Inferences regarding changes over time within each condition were drawn from Dunnett's multiple comparisons test versus baseline, with reported *p* values and confidence intervals adjusted for multiplicity. Inferences regarding the differences between groups were drawn from Šidák's Multiple Comparisons Test, restricted to comparisons at the same timepoint between groups. Inferences regarding the differences between groups were drawn from Šidák's Multiple Comparisons Test, applied to the change from baseline and restricted to comparisons at the same timepoint between groups.

## 3 RESULTS

### 3.1 Participant characteristics

Table 1 shows participant characteristics, including anthropometric data and results from the graded exercise test. All participants were experienced with cycling, had high levels of aerobic fitness, and identified as triathletes, runners, or cyclists. Since the intent of the study was to identify the impact of blockade on %SmO_2_ zero‐slope and time to task failure across populations, and not as a function of biological sex, all data for male and female participants are combined.

### 3.2 Time to task failure and estimated zero‐slope power

**FIGURE 2:**
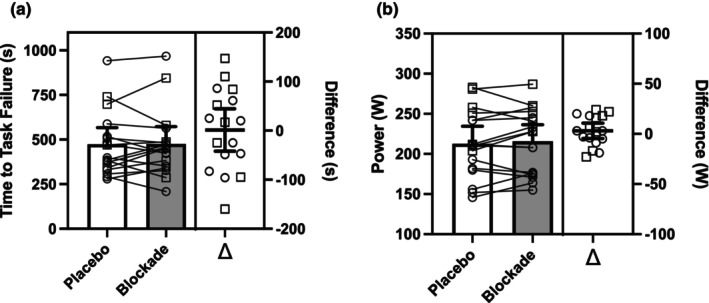
Estimation plots showing time to task failure in the severe domain (a) and estimated zero slope power (b) in placebo (open bars) and antihistamine (closed bars) conditions. Left panel shows group averages and individual points, and right panel shows the difference between conditions (Δ, blockade minus placebo). (○) indicate females and (□) indicate males. Data are means and 95% CI, *n* = 17. There were no differences between conditions (blockade vs. placebo).

The estimated power at zero slope and time to task failure are shown in Figure 2. Estimated zero‐slope was not different (*p* = 0.41) between placebo (213 [191, 234] W) and blockade (216 [195, 236] W) conditions. During the final stage, the time to task failure was not different (*p* = 0.95) between placebo (473 [380, 566] s) and blockade (474 [377, 572] s) conditions. The final stage elicited a negative %SmO_2_ slope in both placebo (−1.7 [−1.2, −2.2] %) and blockade (−1.4 [−0.9, −2.0] %) conditions, but there was no difference between the conditions (*p* = 0.83).

### 3.3 Tissue oxygenation and SmO_2_ slope

**FIGURE 3:**
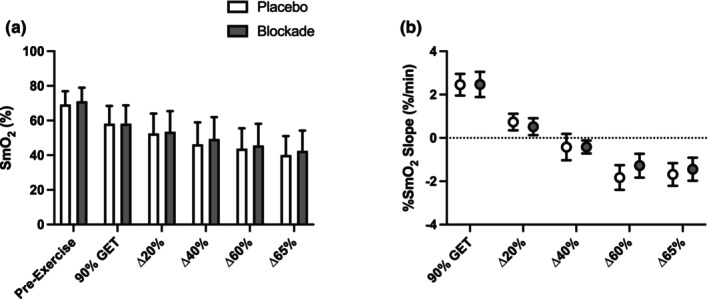
Tissue oxygenation (a) and slopes for each stage (b) in placebo (open bars) and blockade (closed bars) conditions. Tissue oxygenation was the average across the final minute of the stage. Both measurements showed a main effect of stage (*p* <0.05), but there was no difference between conditions (blockade vs. placebo). GET, gas exchange threshold. Data are mean and 95% CI, *n* = 17.

Tissue oxygenation and %SmO_2_ slopes throughout the stages are displayed in Figure 3. %SmO_2_ slope decreased as the intensity increased (main effect of stage; *p <* 0.05), indicating the selected intensities spanned the moderate, heavy, and severe domains. Stage slopes were positive in the predicted moderate domain, approached zero in the predicted heavy domain, and were increasingly negative in the predicted severe domain. However, %SmO_2_ slopes for each stage were not different between conditions (*p* = 0.43). Tissue oxygenation showed a main effect of stage (i.e., lower with higher intensities; *p* < 0.05) but was not different between placebo and blockade (*p* = 0.46) (Figure 3). Tissue oxygenation was lowest in the time to task failure trial but was not different between placebo and blockade.

### 3.4 Gas exchange, heart rate, and rating of perceived exertion

**FIGURE 4:**
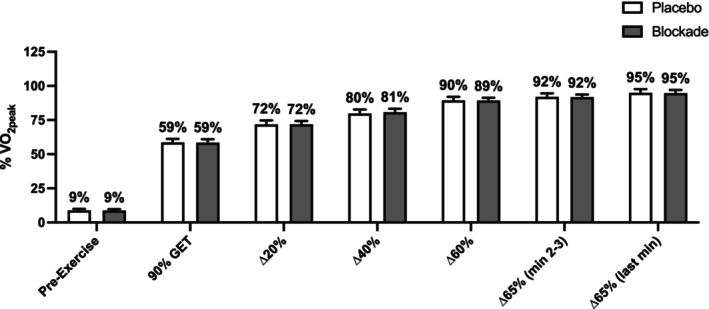
VO_2_ as a percentage of VO_2peak_ in placebo (open bars) and blockade (closed bars) conditions. VO_2_ as a percentage of VO_2peak_ showed a main effect of the stage (*p* < 0.05), but there was no difference between conditions (blockade vs. placebo). GET, gas exchange threshold. Data are means and 95% CI, *n* = 17.

During the staged exercise tests, VO_2_, VCO_2_, respiratory exchange ratio, heart rate, and rating of perceived exertion all showed a main effect of stage (*p* < 0.01) but were not different between conditions (Table 2). VO_2_ as a percentage of VO_2peak_ increased throughout the stages (*p* < 0.05, Figure 4) but was not different between conditions (*p* = 0.95). During the final minute of the time to task failure trial, VO_2_ was 3.77 [3.38, 4.17] L/min and 3.77 [3.37, 4.16] L/min, VCO_2_ was 3.65 [3.26, 4.03] L/min and 3.64 [3.25, 4.03] L/min, and respiratory exchange ratio was 0.97 [0.95, 0.99] and 0.97 [0.94, 1.00] in placebo and blockade, respectively.

**TABLE 2:** Stages of protocol.

		Pre‐exercise	90% GET	∆20%	∆40%	∆60%
VO_2_ (L/min)	Placebo	0.36 [0.31, 0.40]	2.32 [2.11, 2.53]^a^	2.84 [2.57, 3.11]^a^	3.16 [2.86, 3.47]^a^	3.55 [3.20, 3.91]^a^
	Blockade	0.35 [0.31, 0.39]	2.31 [2.11, 2.51]^a^	2.84 [2.59, 3.09]^a^	3.20 [2.90, 3.50]^a^	3.54 [3.20, 3.89]^a^
VCO_2_ (L/min)	Placebo	0.34 [0.28, 0.39]	1.85 [1.64, 2.07]^a^	2.56 [2.32, 2.81]^a^	2.96 [2.68, 3.23]^a^	3.48 [3.13, 3.84]^a^
	Blockade	0.32 [0.29, 0.35]	1.93 [1.77, 2.10]^a^	2.55 [2.33, 2.78]^a^	3.02 [2.72, 3.31]^a^	3.48 [3.14, 3.83]^a^
Respiratory exchange ratio	Placebo	0.93 [0.88, 0.99]	0.84 [0.81, 0.87]^a^	0.90 [0.87, 0.93]^a^	0.94 [0.91, 0.96]^a^	0.98 [0.95, 1.01]^a^
	Blockade	0.93 [0.89, 0.97]	0.84 [0.82, 0.86]^a^	0.90 [0.88, 0.93]^a^	0.94 [0.92, 0.97]^a^	0.98 [0.96, 1.01]^a^
Heart rate (beats/min)	Placebo	76 [69, 84]	136 [128, 145]^a^	152 [144, 160]^a^	162 [154, 169]^a^	171 [164, 178]^a^
	Blockade	75 [66, 85]	138 [129, 147]^a^	154 [146, 162]^a^	164 [157, 172]^a^	172 [166, 179]^a^
Rating of perceived exertion	Placebo	6 [6, 6]	9 [8, 10]^a^	12 [11, 12]^a^	13 [12, 14]^a^	15 [14, 16]^a^
	Blockade	6 [6, 6]	9 [8, 10]^a^	12 [11, 12]^a^	13 [12, 14]^a^	16 [14, 17]^a^

*Note*: VO_2_, VCO_2_, and respiratory exchange ratio are averaged over the final minute of each stage. Heart rate and rating of perceived exertion were recorded during the last 30 seconds of each stage. Data are means [95% CI]. There were no differences between conditions for any timepoint.

^a^ *p* <0.001 versus pre‐exercise.

**FIGURE 5:**
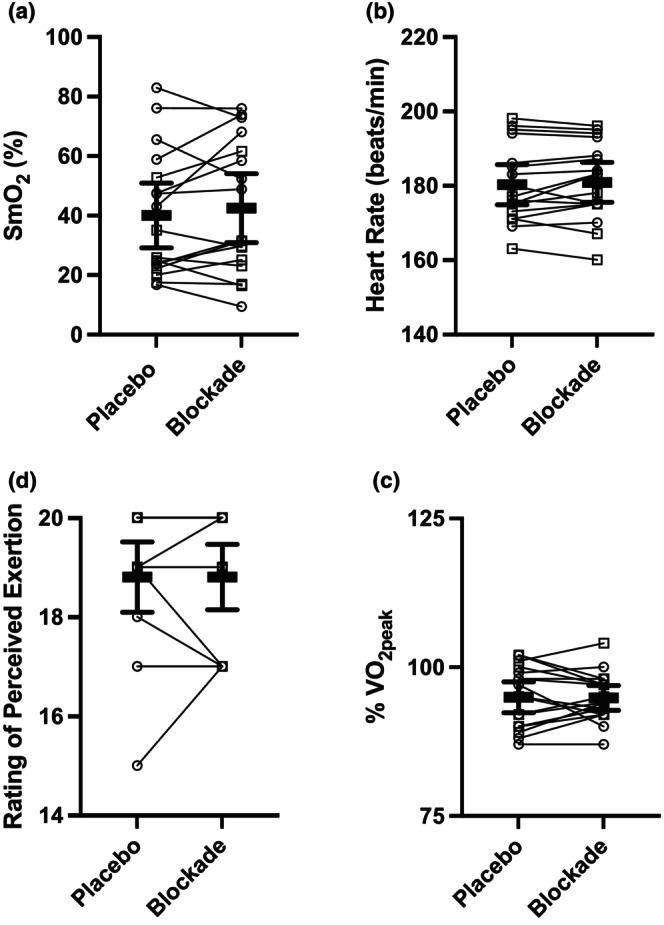
Tissue oxygenation (a), peak heart rate (b), VO_2_ as %VO_2peak_ (c), rating of perceived exertion (d) during the time to task failure trial in placebo (open bars) and blockade (closed bars) conditions. %SmO_2_ and %VO_2peak_ are averaged over the final minute. Heart rate is the highest heart rate achieved during the time to task failure trial, and a rating of perceived exertion was taken at task failure. (○) indicates females and (□) indicates males. There were no differences between blockade and placebo. Data are means and 95% CI, *n* = 17.

Tissue oxygenation, heart rate, rating of perceived exertion, and the percentage of VO_2peak_ during the final minute of the time to task failure trial are presented in Figure 5. During the final stage, VO_2_ increased from 92 [90, 95]% and 92 [90, 94]% VO_2peak_ during minute three to 95 [92, 98]% and 95 [97, 93]% VO_2peak_ during the final minute, following placebo (*p* = 0.04) and blockade (*p* = 0.02), respectively. Heart rate was highest during the final stage and was 180 [175, 186] bpm and 181 [176, 186] bpm for placebo and blockade conditions. These corresponded with 99% [98, 100] (placebo) and 100% [98, 101] (blockade) of the peak heart rate achieved during the graded exercise test. Rating of perceived exertion was highest at the end of the final stage (task failure) and was 19 [18, 20] with placebo and 19 [18, 19] with blockade.

## 4 DISCUSSION

Histamine‐receptor blockade does not appear to alter oxygen supply–demand balance or the power associated with the maximal metabolic steady state (i.e., heavy‐severe domain boundary). Additionally, histamine‐receptor blockade does not alter time to task failure in the severe domain. These results indicate that histamine‐receptor blockade does not impact the external workload at which the maximal metabolic steady state occurs, nor does it impact relatively shorter, high‐intensity exercise performance.

### 4.1 Histamine‐receptor blockade and oxygen supply–demand balance

Given the prominent role of histamine in sustained postexercise vasodilation (Barrett‐O'Keefe et al., 2013; McCord et al., 2006; McCord & Halliwill, 2006) it was plausible that blockade would reduce blood flow to exercising tissues. However, during dynamic knee extension, histamine‐receptor blockade increases femoral blood flow and vascular conductance while also decreasing intramuscular pH (Ely et al., 2020). This finding pointed toward a “histamine paradox” such that during exercise, blocking histamine receptors appears to increase bulk blood flow in conduit vessels but may impair muscle perfusion (i.e., greater muscle tissue acidity and decreased performance). Skeletal muscle is a heterogeneous and complex tissue, and blood flow distribution within exercising tissue is not uniform. Within skeletal muscle, tissue metabolism and vascular smooth muscle constitute a local control system tightly coupling blood flow to tissue metabolic requirements (Laughlin et al., 2012). Histamine acts on vascular smooth muscle, and its blockade may dysregulate the coupling of local perfusion and tissue metabolic requirements, potentially impairing oxygen supply–demand matching during exercise and lowering the intensity at which the maximal metabolic steady state occurs, that is, the heavy‐severe domain boundary.

Evaluating dynamic changes in muscle oxygenation through %SmO_2_ slope provides real‐time insight into oxygen supply–demand balance during exercise. The highest external work rate at which oxygen supply can meet demand (i.e., zero‐slope) indicates the boundary between the heavy‐and severe domains (Kirby et al., 2021; Matthews et al., 2023; Wilkins et al., 2025). At intensities above this boundary, there is an inability of perfusion to meet metabolic demand, leading to an increase in oxygen uptake and the accumulation of metabolites, necessitating either a reduction in work rate or the cessation of exercise (Jones et al., 2010, 2019; Matthews et al., 2023). Dysregulation of intramuscular perfusion may lower this boundary, providing insight into the previously seen increase in metabolite production despite increases in bulk blood flow. Further, a lowering of the heavy‐severe domain boundary may reduce work capacity, explaining the impaired exercise performance following histamine‐receptor blockade (Ely et al., 2019).

The present results suggest that histamine‐receptor blockade does not impact the ability to balance oxygen supply and demand or the highest external work rate at which this can be achieved (i.e., heavy‐severe domain boundary). Thus, it is unlikely that the previously reported ergolytic effect of histamine‐receptor blockade (Ely et al., 2019) is due to an inability to match oxygen supply and demand or a reduction in the power eliciting the maximal metabolic steady state.

### 4.2 Histamine‐receptor blockade and cycling performance

Contrary to previous findings, we found no effect of blockade on our performance metric, time to task failure in the severe domain. The previous investigation used a 10‐km cycling time trial as its metric and found that blockade slowed completion by approximately 1% (Ely et al., 2019). We posit several potential explanations for the differing impact of histamine‐receptor blockade on performance metrics between the two studies, such as the differences in the duration of the performance test, the presence or absence of preceding exercise and self‐pacing, and potential histamine‐mediated changes in nutrient delivery or metabolite washout.

### 4.3 Duration

It may be that the impact of antihistamines becomes more pronounced as the duration of exercise increases. There is an appreciable difference in the duration of exercise between the investigations. The measurement of performance in the current investigation, time to task failure, lasted roughly half as long as the measurement of performance in the previous investigation (~475 s vs. ~1000 s, respectively). Additionally, the previous investigation employed 2 h of cycling at 50% VO_2peak_ before the 10‐km time trial, whereas in this investigation, the participants performed approximately 43 min of exercise prior to the time‐to‐exhaustion trial. Prolonged exercise in the moderate domain has been shown to impact physiological responses at exercise intensity transitions (Gallo et al., 2024; Stevenson et al., 2022). Histamine‐receptor blockade may impact these transitions during extended exercise, having knock‐on effects for subsequent exercise performance. It has been suggested that histamine‐receptor blockade impacts nutrient delivery and metabolite washout (Ely et al., 2020), an effect that might be less apparent in shorter durations of exercise where nutrient delivery and metabolite washout are less likely to be limiting factors. Thus, the longer duration of both the performance task and the prior exercise in the previous investigation may have unmasked the effect of histamine‐receptor blockade.

Other factors, related to the performance task, may have led to the differing effects of histamine‐receptor blockade observed in the studies. First, the time taken to complete the 10‐km time trial in the previous investigation (~1000 s) and the time to task failure (~475 s) in the present investigation. Blood lactate concentrations from the previous investigation (Ely et al., 2019) and the VO_2_ and %SmO_2_ slope responses in the present investigation suggest both tasks consisted of significant work performed in the severe domain. Given the relatively similar fitness levels and training histories of the populations, it is likely that the constant work rate trial in the present investigation was performed at an intensity that was further above the heavy‐severe domain boundary. Thus, these findings suggest histamine‐receptor blockade does not impact shorter, high‐intensity efforts. These findings are seemingly in line with previous investigations into only H_1_ receptor blockade, showing that it does not impact muscle strength (Montgomery & Deuster, 1991) or high‐intensity exercise performance (Montgomery & Deuster, 1992). Histamine‐receptor blockade may have a more pronounced effect in events lasting >12 min (e.g., 5 km running and above) than in shorter events, such as those employed in the current investigation.

Two more factors warrant consideration as to the disparate findings surrounding the impact of histamine‐receptor blockade on performance. It has been hypothesized that the use of histamine‐receptor blockade may impact nutrient delivery or metabolite washout, potentially explaining the decreased pH during knee extension with blockade (Ely et al., 2020). Rodent studies show that histamine‐receptor blockade decreases intramuscular glycogen following a walking test (Niijima‐Yaoita et al., 2012). While the effects of blockade on muscle glycogen content in humans have not been studied at this time, histamine‐receptor blockade reduces postexercise interstitial glucose concentrations (Pellinger et al., 2010), insulin sensitivity (Emhoff et al., 2011), and glycogen resynthesis (Van Der Stede et al., 2025) presumably through impairments in glucose delivery. While glycogen depletion is not likely to limit exercise of this duration, alterations in blood flow may reduce metabolite washout, which certainly may impair exercise response. Further, given the impact of blockade on glucose delivery following exercise, it is possible that it impacts nutrient delivery during exercise as well. While the current trial may not have been long enough to be influenced by nutrient delivery, it is not without merit to investigate the effects of histamine‐receptor blockade on nutrient delivery in longer‐duration exercise.

### 4.4 Employing a noninvasive, nonexhaustive protocol to evaluate maximal metabolic steady state

The traditional methodologies for determining the heavy‐severe domain boundary require multiple testing days (Jones et al., 2010) or exhaustive exercise trials (Burnley et al., 2006; Vanhatalo et al., 2007). We employed the zero‐slope protocol, a newly established methodology for determining power at the maximal metabolic steady state, in addressing an interventional question. The zero‐slope protocol uses NIRS to assess dynamic changes in %SmO_2_ over time (i.e., %SmO_2_ slope), indicating the ability to balance oxygen supply and demand in working tissue. This methodology accurately identifies exercise intensity domains above and below the maximal metabolic steady state (Kirby et al., 2021; Matthews et al., 2023; Wilkins et al., 2025), aligns with traditional indicators of exercise intensity (e.g., lactate (Batterson et al., 2023; Matthews et al., 2023) and oxygen uptake (Kirby et al., 2021; Matthews et al., 2023)), and has excellent reliability (ICC > 0.9) and low measurement error (<2%) for predicting power at the heavy‐severe domain boundary for both men and women (Hudgins et al., n.d.). As the methodology relies on intermittent submaximal exercise, it broadens the populations in which it can be employed (i.e., those that may struggle with exhaustive exercise can complete this test) and can also be employed in conjunction with tests of exercise performance, as in the current study.

## 5 CONCLUSION

The primary and secondary aims of this project were to evaluate the impact of histamine‐receptor blockade on oxygen supply–demand balance and heavy‐severe domain boundary, and time to task failure in the severe domain. Compared to placebo, blockade did not alter the oxygen supply–demand balance or the heavy‐severe domain boundary and did not impact time to task failure in the severe domain. Thus, histamine‐receptor blockade does not appear to critically alter the maximal metabolic steady state or performance lasting 6–8 min in the severe domain. While histamine‐receptor blockade appears not to influence relatively short‐duration, high‐intensity exercise, its impact on longer‐duration, variable‐intensity exercise warrants future investigation.

## AUTHOR CONTRIBUTIONS

KSSA, CTM, BWW, and JRH conceived and designed the experiment. KSSA and ISV performed experiments. KSSA, JHH, and ISV analyzed data. KSSA, JHH, CTM, BWW, and JRH interpreted the results of experiments. KSSA and JRH prepared figures and tables. KSSA and JRH drafted the manuscript. All authors edited or revised the manuscript. All authors approved the final version of the manuscript.

## FUNDING INFORMATION

This research was supported by the National Institutes of Health grant R01 AG072805, the Wu Tsai Human Performance Alliance and the Joe and Clara Tsai Foundation, and the Eugene and Clarissa Evonuk Memorial Graduate Fellowship.

## CONFLICT OF INTEREST STATEMENT

The authors have no conflicts of interest, financial or otherwise, that would be affected by the outcome of this publication.

## ETHICS STATEMENT

The Institutional Review Board at the University of Oregon approved this study. Written informed consent was obtained from all participants, and the study conformed to the principles outlined in the Declaration of Helsinki.

## Data Availability

Source data for this study are not publicly available due to privacy or ethical restrictions. The source data are available to verified researchers upon request by contacting the corresponding author.
